# SoMarker: a genetic marker searching tool for *Caenorhabditis elegans*

**DOI:** 10.1093/g3journal/jkae197

**Published:** 2024-08-17

**Authors:** Chuan-Yang Dai, Haobo Zhang, Steven Zuryn

**Affiliations:** Clem Jones Centre for Ageing Dementia Research, Queensland Brain Institute, The University of Queensland, Brisbane, Queensland, 4072, Australia; Independent Scholar, Shanghai, China; Clem Jones Centre for Ageing Dementia Research, Queensland Brain Institute, The University of Queensland, Brisbane, Queensland, 4072, Australia

**Keywords:** *C. elegans*, genetic cross, gene, genetic marker, Java software

## Abstract

*Caenorhabditis elegans* is one of the most popular model organisms used to genetically dissect complex biological phenomena. One common technique used routinely in the *C. elegans* laboratory is the generation of strains carrying combinations of genetic mutations via classical genetic crosses. Here, we have developed a simple and convenient application to quickly identify useful genetic markers (phenotypical and fluorescent) and their chromosomal positions to aid in the development of genetic cross strategies. The user-friendly software identifies and prioritizes markers with the least genetic distance to a gene of interest, as well as displays the strain name, ease of scoring, nature of the marker (fluorescent transgene or phenotypic information), mating efficiency, and number of available alleles. In addition, recombination frequencies between the gene of interest and each genetic marker are calculated automatically. The application, called “SoMarker,” is designed for both MacOS and Windows environments and is available to freely download and modify through open-source software.

## Introduction

**Fig. 1.**
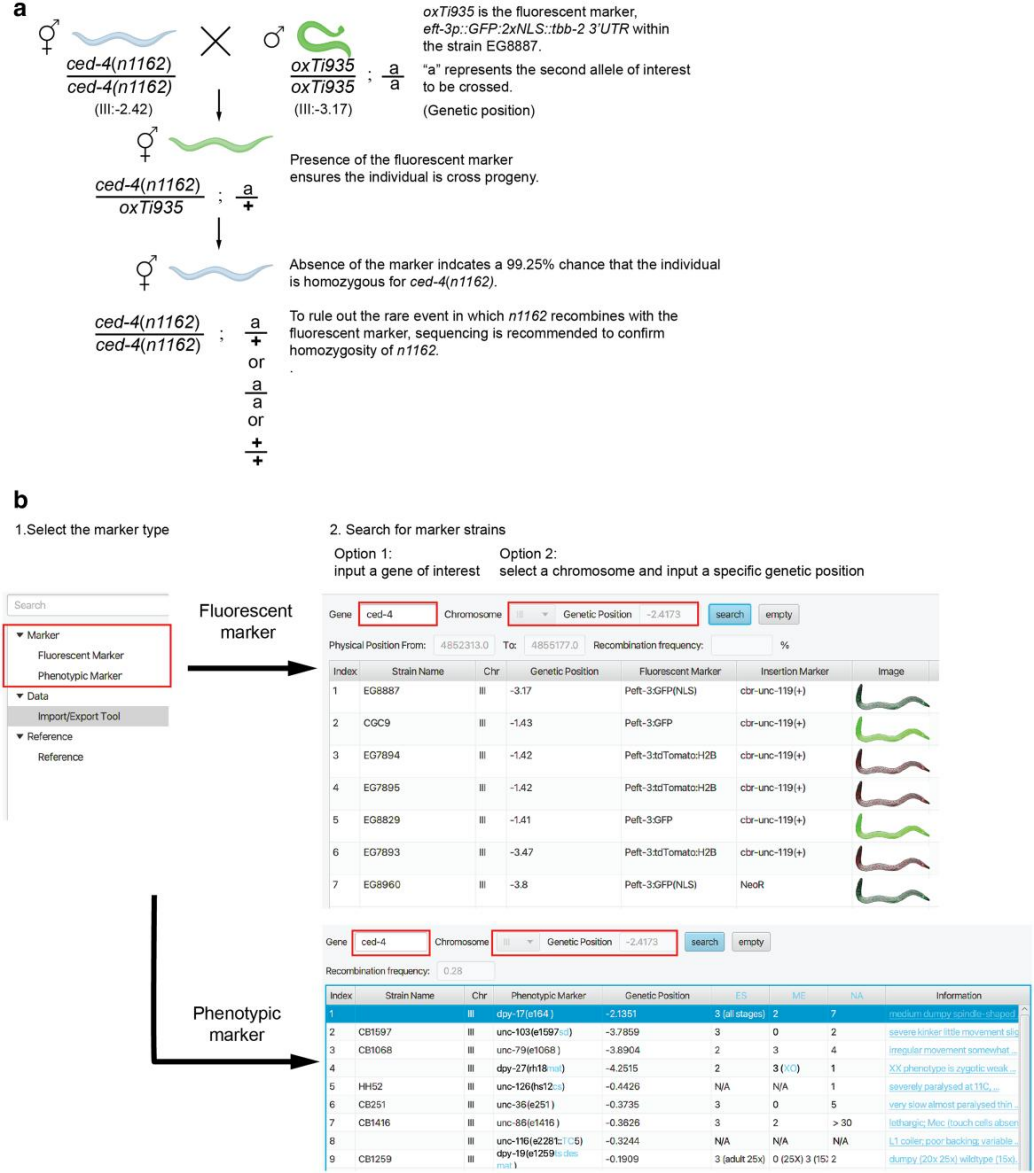
SoMarker can be used to identify useful markers for genetic crosses in *C. elegans*. a) A schematic of a genetic cross where a genetic marker strain is used to ensure the genotype of progeny. The selection marker allele (*unc-119(ed3)* and +*Cbr-unc-119(+)*) used in generating the strain EG8887 has been omitted from this crossing diagram for simplicity, but its segregation may be important for specific experiments. Refer to Fay (2013) on how to set up a cross using genetic markers. Figure was made in BioRender.com. b) A step-by-step instruction of using SoMarker application to search for fluorescent marker strains and phenotypic marker strains useful for *ced*-*4*. The recombination frequency between *ced-4* and each selected marker is displayed. Maker strains can also be searched by inputting a specific chromosome and genetic position.

Genetic crosses are performed daily in *C. elegans* laboratories, allowing the generation of animals carrying combinations of genetic mutations. To determine the presence of a specific mutation in cross progeny, genotyping is typically used for alleles that do not present an obvious phenotype. However, for those superficially wild-type animals carrying single-nucleotide substitutions or small insertions/deletions, genotyping is less straightforward and may require sequencing. Therefore, visible genetic markers are often used within cross strategies to follow difficult to genotype alleles. Genetic markers are genetically linked to the mutation of interest due to their close physical proximity on the chromosome. As such, they can be used to ensure the presence of an allele during the cross and after its completion by following the presence and then the absence of the phenotypic or fluorescent marker (Fig. 1a) (Riddle *et al*. 1997; Fay 2013; Frøkjær-Jensen *et al*. 2014).

To identify an appropriate phenotypic or fluorescent genetic marker, the investigator must manually source the genetic position of candidate markers from either the book *C. elegans* II (2nd edition) (Riddle *et al*. 1997) or the website Wormbuilder (wormbuilder.org) (Frøkjær-Jensen *et al*. 2014), respectively. The process of identifying and simultaneously comparing multiple genetic markers is therefore currently suboptimal. Thus, we developed a simple Java-based software, SoMarker, to facilitate the search and selection process. SoMarker is an open-source, user-friendly software, which automatically identifies the top 20 genetic markers (either phenotypical or fluorescent marker) sorted in ascending order by genetic distance to the gene or chromosomal location of interest. Furthermore, it provides the calculation of the recombination frequency, which is calculated by the absolute value of the difference between the genetic positions of the gene of interest and the genetic markers identified, as well as information about these markers. These features of the application significantly streamline the process of searching for genetic marker strains.

## Results

As an example, to identify a suitable crossing marker for the superficially wild-type *ced-4*(*n1162*) point mutation (Borsos *et al*. 2011), open the SoMarker application and select either “Fluorescent Marker” or “Phenotypic Marker” under the “Marker” tab on the left panel. Next, enter the name of the gene, “*ced-4*,” in the text box labeled “Gene” located at the top of the window (Fig. 1b). Click on the “Search” button, and a list of genetic markers and their information is displayed in ascending order of genetic proximity to *ced-4* (Fig. 1b). Information for each genetic marker includes the strain name harboring the marker, representative schematic images of the visible fluorescent transgene in the “Fluorescent Marker” interface, and ease of scoring (ES), mating efficiency (ME), number of alleles (NA), and phenotypic information in the “Phenotypic Marker” interface (Fig. 1b). The recombination frequency between the genetic marker and gene of interest is calculated when one of the markers is selected (Fig. 1b). Alternative to the name of a gene, a chromosome and genetic position can be entered into SoMarker to identify markers, which may be useful in cases where the name of a gene has changed.

**Fig. 2.**
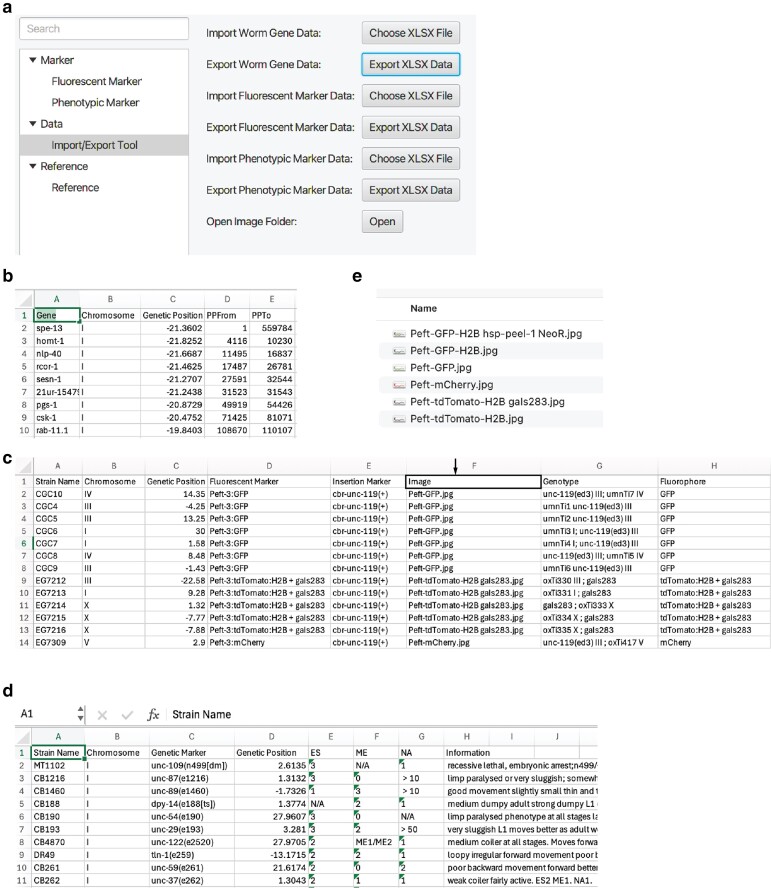
The edit function of SoMarker can streamline the process of updating gene and marker information. a) The interface of the edit function that allows the export of editable gene and marker information in a .xlsx format. b) The spreadsheet of gene information. PPFrom, physical position from; PPTo, physical position to. c) The spreadsheet of fluorescent marker information. The box indicated by the arrow shows the “Image” column used to edit images in SoMarker. d) The spreadsheet of phenotypic marker information. e) The directory of worm images.

The information in SoMarker was retrieved from WormBase (https://wormbase.org/) and based on the completed genome sequence (PRJNA13758). All strains listed in SoMArker are available from the Caenorhabditis Genetics Center (CGC). Briefly, the fluorescent marker strains included in SoMarker were generated by *miniMos* transposable element with a selection marker of either *unc-119* rescue or antibiotic resistance (Frøkjær-Jensen *et al*. 2014). SoMarker was designed to be compatible with both Windows and MacOS environments and the application and the source code can be obtained from GitHub (https://github.com/zhb1991nm/SoMarker/releases/tag/v0.1.0). Due to its open source, the software can be modified and updated as users see fit. However, to easily facilitate the addition of genetic markers and edit gene information, the software includes import and export functions under the “Data” tab (Fig. 2a). Briefly, by clicking on the “Export XLSX Data” button (Fig. 2a) next to “Export Worm Gene Data,” a spreadsheet will be generated and saved to a directory of choice (Fig. 2b). Information can be added, removed, or edited on this spreadsheet and imported back into the software by clicking on the “Choose XLSX File” button next to “Import Worm Gene Data” (Fig. 2a). The same can also be achieved for both fluorescent marker (Fig. 2c) and phenotypic marker (Fig. 2d) information by following the same process, but instead clicking on the buttons corresponding to each category in the “Data” tab (Fig. 2a). Finally, new worm images can be added or modified to fluorescent marker information by clicking on the “Open” button next to “Open Image Folder” (under the “Data” tab) and copying a new image to this folder (Fig. 2e). To link the image with a particular fluorescent marker within SoMarker, add the file name (including the file extension name) to the “Image” column in the fluorescent marker spreadsheet (Fig. 2c; see instructions above for accessing this spreadsheet) for the appropriate strain. This new image will be updated in SoMarker after the modified spreadsheet is imported into the software (see instructions above).

## Data Availability

The application and the source code can be obtained from GitHub (https://github.com/zhb1991nm/SoMarker).
